# Lichen with triple involvement: cutaneous, mucous and phanerian

**DOI:** 10.11604/pamj.2018.31.12.16510

**Published:** 2018-09-04

**Authors:** Mohamed El Amraoui, Badredine Hassam

**Affiliations:** 1 Dermatology Department, Avicenna Hospital, Ibn Sina University Hospital, Rabat, Maroc

**Keywords:** Lichen, cutaneous, mucous, phanerian, involvement

## Image in medicine

Lichen is a common chronic inflammatory dermatitis that can affect the skin, mucous membranes and phaneres. The simultaneous involvement of the three compartments is rare and testifies to the severity and activity of the disease. We present a case of this triple involvement. However, the complete muco-cutaneous and phanerienic forms are rare, testify to the severity of the disease and justify a systemic treatment and a regular and long-term monitoring view the risk of degeneration of the mucous lesions. We report the case of this 60-old-man, followed for papular dermatosis, hyperpigmentary, very itchy, generalized, with involvement of the genital mucosa, in the form of erosive balanitis and nails of the hands and feet. The cutaneous histology was in favor of lichen. The biological examinations did not show any dysthyroidism, liver or autoimmune diseases. The patient was placed on systemic and topical corticosteroids. A pigmentogenic evolution of the cutaneous lesions, a beginning of bleaching of the nail lesions and a resistance of the genital lesions were noted.

**Figure 1:**
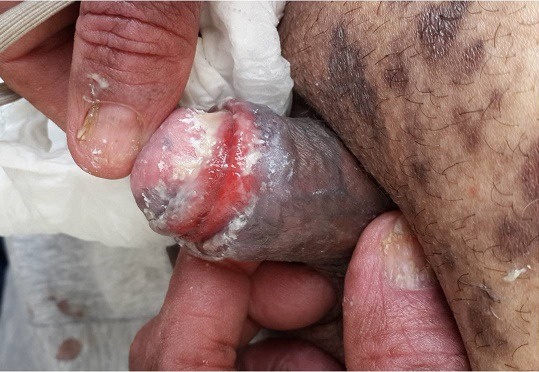
Lichen with triple involvement: cutaneous, mucous and phanerian

